# Occult solitary submucosal jejunal metastasis from esophageal carcinoma

**DOI:** 10.1186/1477-7819-3-44

**Published:** 2005-07-16

**Authors:** Joerg Lindenmann, Franz Gollowitsch, Veronika Matzi, Christian Porubsky, Alfred Maier, Freyja Maria Smolle-Juettner

**Affiliations:** 1 Department of Thoracic and Hyperbaric Surgery, University Medical School, Auenbruggerplatz 29, Graz, Austria; 2 Department of Pathology, University Medical School, Auenbruggerplatz 35, Graz, Austria

## Abstract

**Background:**

Metastatic tumors of the intestinal tract from extra-abdominal sites are rare. In esophageal cancer, the liver, lung and the bones are the most common sites of metastases. Metastasis to intestines are very rare.

**Case presentation:**

A 54-year old male was admitted with esophageal squamous cell carcinoma (SCC) associated with dysphagia II-III and weight loss of 20 kg. Preoperative routine staging failed to detect any metastases. A transthoracic esophagectomy and orthotopic gastric pull-up with collar esophago-gastrostomy, associated with 2-field lymphadenectomy was perfromed. During the digital placement of the naso-jejunal feeding catheter a submucosal jejunal nodule with a diameter of 1 cm, about 40 cm distal to the duodeno-jejunal fold was detected which was completely resected by jejunotomy. Histopathology of jejunal nodule showed metastasis from esophageal squamous cell carcinoma.

**Conclusion:**

Because of the extensic esophageal lymphatic system, an occult widespread dissemination of the tumor cells into the abdominal cavity is possible. Additional intraoperative evaluation of the small intestine and the complete abdominal cavity should be performed in every operation of esophageal carcinoma to detect possible occult intraabdominal metastases.

## Background

Metastatic tumors of the intestinal tract from extra-abdominal sites are rare. In esophageal cancer, the liver, lung and the bones are the most common sites of metastases. Metastases to the small intestine are very rare. We describe the case of a 54-year-old man suffering from esophageal carcinoma, who underwent transthoracic esophagectomy and 2-field lymphadenectomy, the reconstruction was done by orthotopic gastric pull-up. During the operation, a solitary submucosal jejunal metastasis of the esophageal carcinoma was detected and excised.

## Case presentation

A 54-year-old male, alcohol and tobacco user, was admitted with squamous cell carcinoma of the esophagus associated with the clinical symptoms of high-grade dysphagia and weight loss of 20 kg. Routine staging was done by computerized tomography (CT)-scan of the thorax and abdomen, ultrasonography, positron emission tomography (PET) scan, esophago-gastro-duodenoscopy and bronchoscopy. Functional evaluation was done by electrocardiogram (ECG), cardiac ultrasonography and spiroergometry.

The CT-scan of the thorax and the mediastinum showed a tumor of the middle third of the esophagus with a suspicion of tumor infiltration of the thoracic aorta and the left main bronchus. However, infiltration of the main bronchus could be excluded by bronchoscopy. Infiltration of the thoracic aorta could also be excluded by MRI-angiography. The CT-scan of the abdomen and the abdominal ultrasound showed no signs of tumor spread. The PET-scan demonstrated a pathological tracer uptake at the level of the middle third of the esophagus with no signs of distal metastasis. Esophago-gastro-duodenoscopy showed a tumor stenosis of 7 cm length, from 30 to 37 cm, and a diameter of 4 – 6 mm, further there was an intraluminal obstruction about more than 50 % which was additional confirmed by an esophagogram.

Transthoracic esophagectomy and 2-field lymphadenectomy was carried out as reported in literature [1,2]. Intraoperative frozen section showed no residual tumor at the lateral, distal and proximal margins. The mediastinal lymph nodes showed no signs of tumor infiltration. Reconstruction was done by orthotopic gastric pull-up and hand-sewn side to end collar esophago-gastrostomy. In order to perform postoperative early feeding a naso-jejunal catheter was introduced and placed distal to the duodeno-jejunal fold.

During the digital placement of the naso-jejunal feeding catheter a submucosal jejunal nodule with a diameter of 1 cm was detected about 40 cm distal to the duodeno-jejunal fold. The nodule was completely resected by jejunotomy. Intraoperative frozen section showed a submucosal metastasis of the esophagus. Further evaluation of the small intestine and the complete abdominal cavity showed no signs of metastasis.

**Figure 1:**
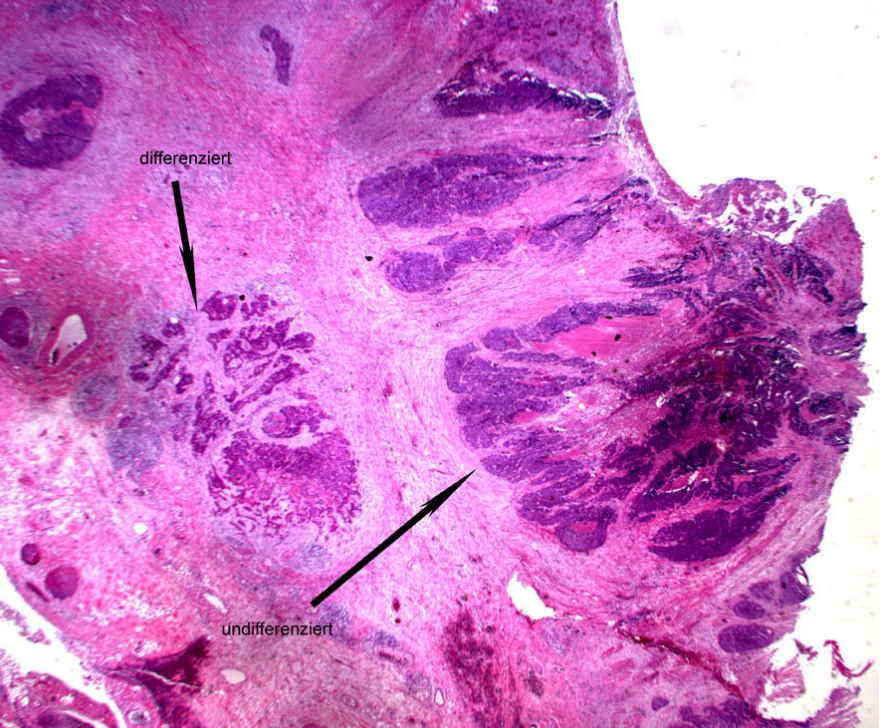
Photomicrograph of specimen showing the esophageal SCC (hematoxylin and eosin × 25).

**Figure 2:**
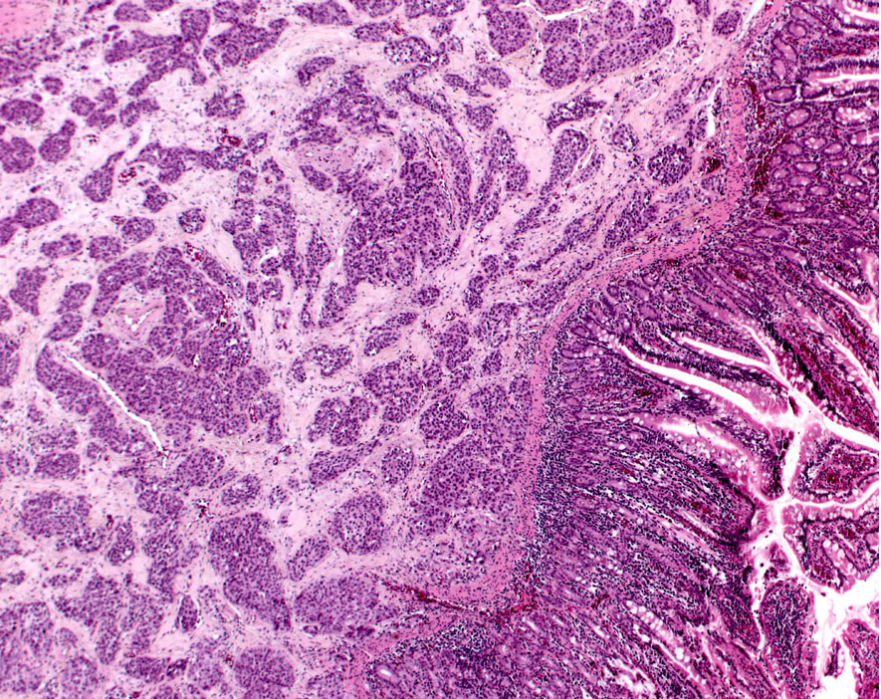
**Submucosal jejunal metastasis of the esophageal squamous cell carcinoma (SCC). **Photomicrograph of specimen showing the metastasis, associated with local submucosal venous and lymphatic infiltration. Neither the jejunal serosa nor the mucosa are affected by the carcinoma (hematoxylin and eosin × 50).

Finally histopathological work-up of the specimens confirmed the diagnosis of squamous cell carcinoma of the esophagus (Figure 1) and a solitary submucosal jejunal metastasis. Furthermore, the submucosal jejunal metastasis was associated with local submucosal venous and lymphatic infiltration (Figure 2). In immunhistochemical tests the tumor cells showed reaction to CEA and CK 5–6. The definitive staging was T_3_, N_0_, M_1_, L_1_, G_3_, R_0_.

The further course of the patient was uneventful and he could be discharged on the 14^th ^postoperative day. As this was stage IV squamous cell carcinoma of the esophagus adjuvant chemotherapy was not offered to the patient [3]. However, a follow-up protocol was initiated with CT-scans every 3-month for the first year.

## Discussion

Metastatic tumors of the intestinal tract from extra- abdominal sites are rare [4,5]. In esophageal cancer, the liver, lung and the bones are the most common sites of metastases. Metastases to the small intestine are very rare [6].

Squamous cell carcinoma of the esophagus is characterized by an extensive lymphatic dissemination via the longitudinal lymphatic system along the esophagus, which drains in to a large number of widespread lymph nodes of the thorax and the abdomen depending on the location of the tumor [2]. Retrograde spread to the cervical, mesenteric and iliac lymph nodes are very unusual, but have been reported in literature [7,8]. The intraabdominal region can be reached via the vertebral venous system [9,10]. This huge and widespread lymphatic and hematogenous system connecting the esophagus with the intraabdominal region may be sufficient to explain, why this small solitary metastasis of the jejunum was discovered for the first time during the operation and could probably be responsible for this extraordinary bad prognosis in patients with esophageal squamous cell carcinoma caused by the occult widespread dissemination of the tumor cells.

In this case, the operation was absolutely indicated to prevent the patient from further progression of his dysphagia, to avoid the development of a malignant esophagotracheal fistula with its typical complication, mainly bronchopneumonia due to nocturnal aspiration and to avoid the development of an jejunal ileus caused by intraluminal obstruction by that solitary jejunal metastasis. A quite interesting aspect is, why this solitary metastasis could not be discovered by the PET-scan, which showed a pathological tracer uptake at the level of the middle third of the esophagus, further an unspecific tracer uptake due to inflammatory disease in the subhepatic region of the right upper abdomen was seen but there was no suspicion of further distal metastases. The main reason for this failure of the PET-scan could be the high incidence of artifacts caused by many different small intraabdominal focuses of inflammation which make the correct assessment of the abdominal cavity quite difficult.

## Conclusion

Additional intraoperative evaluation of the small intestine and the complete abdominal cavity should be performed in every laparotomy for esophageal carcinoma to detect possible occult intraabdominal metastases. PET is not sensitive enough to pick small metastasis.

## Competing interests

The author(s) declare that they have no competing interests.

## Authors' contributions

**JL: **Preparation of draft manuscript, Surgical management, Revision of manuscript and preparation of final manuscript.

**VM: **Preparation of manuscript, surgical management

**AM, **Surgical management, Revision of manuscript and preparation of final manuscript

**CP, **Surgical management

**FG: **Histopathological work-up, immunhistochemical tests, photomicrograph of specimen.

**FMSJ**: Revision of manuscript and preparation of final manuscript.

All authors read and approved the final version of the manuscript.
